# Recent Advances in Renal Denervation Therapy and Novel Applications of High-Intensity Focused Ultrasound

**DOI:** 10.1155/cdr/6692871

**Published:** 2025-08-30

**Authors:** JunHao Wang, Yu Ma, Ximing Li

**Affiliations:** ^1^Department of Thoracic Clinical Medicine, Tianjin Medical University, Tianjin, China; ^2^Department of Cardiology, Tianjin University Chest Hospital, Tianjin, China; ^3^Tianjin Key Laboratory of Cardiovascular Emergency and Critical Care, Tianjin, China

## Abstract

Hypertension constitutes a major risk factor for cardiovascular diseases. Globally, the management and control of hypertension remain suboptimal. At present, pharmacological intervention is a critical strategy for patients with hypertension to achieve blood pressure regulation. Nevertheless, inadequate adherence to prescribed medication significantly impedes effective blood pressure control. Renal denervation (RDN) has emerged as a minimally invasive intervention that has the potential to achieve long-term reduction in blood pressure. Many preclinical and clinical trials have validated the effects of RDN. While trials such as SYMPLICITY HTN-3 raised questions about the efficacy of RDN, the majority of subsequent clinical trials have demonstrated benefits in patients with resistant hypertension. Presently, traditional RDN therapy is characterized by invasiveness and technical complexity, so the utilization of noninvasive high-intensity focused ultrasound (HIFU) has been incorporated into RDN procedures. This review is aimed at consolidating data on the efficacy and safety of various RDN modalities, examining the implementation of noninvasive HIFU in RDN practices, and suggesting potential avenues for future advancements and opportunities.

## 1. Introduction

Hypertension stands as a significant risk factor contributing to overall morbidity and mortality of cardiovascular disease on a global scale. The long-term effects of untreated hypertension on the heart, brain, and kidneys may cause severe health problems like strokes, myocardial infarctions, and renal failure. Globally, 59% of women and 49% of men reported having hypertension in 2019 [1], with approximately 33% of the global population, totaling 8 billion people, affected [2]. In China alone, statistics from 2015 indicated a staggering 245 million cases of hypertension [3]. In response to the growing global burden of hypertension, several international organizations have issued clinical guidelines to improve diagnosis and management.

Two well-established documents from North America and Europe—the 2017 American College of Cardiology (ACC)/American Heart Association (AHA) guideline [4] and the 2018 European Society of Cardiology (ESC)/European Society of Hypertension (ESH) guideline [5]—both provided standardized classifications of hypertension and refined definitions of resistant hypertension (shown in Table 1).

The recommended therapeutic strategy for hypertension should encompass appropriate lifestyle modifications and the administration of optimal or best-tolerated doses of three or more antihypertensive medications, which should include a diuretic, an angiotensin-converting enzyme (ACE) inhibitor or an Angiotensin II receptor blocker (ARB), and a calcium channel blocker (CCB). However, poor adherence to prescribed antihypertensive medications remains a major contributor to suboptimal blood pressure (BP) control, as evidenced by extensive observational studies [6] indicating that over 50% of patients either failed to comply with treatment or exhibited poor adherence [7]. And routine antihypertensive regimens typically have limited efficacy in resistant hypertension. This phenomenon of resistant hypertension is on the rise globally, with prevalence rates ranging from 12% to 18% among patients [8]. In these cases, exploring alternative treatment options may be necessary to effectively manage BP.

Renal denervation (RDN) is a catheter-based procedure—using radiofrequency (RF), ultrasound, or chemical ablation—to disrupt renal sympathetic nerves, thereby lowering sympathetic activity and reducing BP. It has shown promise as an alternative therapy for resistant hypertension, particularly among patients with poor medication adherence. Evidence suggests greater efficacy in treating systolic/diastolic hypertension compared to isolated systolic hypertension [9]. The 2021 ESH supplement recommended RDN as a clinical option for resistant hypertension [10], and a 2023 consensus by the ESH and the European Association of Percutaneous Cardiovascular Interventions (EAPCI) further supported its role in hypertension management [11]. In November 2023, the Paradise RDN system and other RDN devices were approved for clinical use by the US Food and Drug Administration (FDA) [12].

## 2. Anatomy of the Renal Sympathetic Nerves

The renal plexus is comprised of both parasympathetic and sympathetic nerves, which exhibit activity in close proximity to the renal arteries and provide innervation to various structures within the renal parenchyma, including tubular components, vascular elements, and juxtaglomerular granular cells [13]. Research conducted by Mahfoud et al. [14] demonstrated that the greatest concentration of nerves was observed in the proximal and middle segments of the renal artery, with the lowest density found in the distal portion. Furthermore, approximately 90% of these nerves were situated within a distance of 6–7 mm from the lumen of the renal artery. The density of afferent fibers in the sympathetic nerve is significantly lower than that of efferent fibers, with the nerve also enveloping the accessory renal artery. Recent findings indicate that vessels following the bifurcation of the renal artery may present more effective targets for RDN ablation [15].

## 3. Various Techniques for RDN

RDN encompasses multiple interventional techniques developed to disrupt renal sympathetic nerve activity, each employing distinct mechanisms of action. The following summarizes four principal RDN modalities currently under investigation or in clinical application:

1. The RF catheter RDN technology is intended to ablate the nerves located in the adventitia of the renal artery through thermal energy. Utilizing a four-spaced spiral electrode catheter heated by medium frequency alternating current, RDN is a prevalent treatment modality for drug-resistant hypertension. While the renal artery wall can tolerate the thermal energy generated, nerves within a 7 mm radius of the artery lumen are susceptible to damage. Following the exclusion of secondary causes of resistant hypertension [16], RF catheter RDN remains a primary therapeutic option. Currently, RF RDN (e.g., Medtronic's Symplicity Spyral) has received FDA and CE (Conformité Européenne) approval and is clinically adopted in selected centers for patients with uncontrolled or resistant hypertension [17, 18].
2. Ultrasonic-based RDN, utilizing high-frequency ultrasound technology, shows promise in clinical studies demonstrating its efficacy and safety [19]. RDN through intravascular ultrasound catheterization selectively damages renal nerves, leading to a reduction in sympathetic nervous system activity. In comparison to RF methods, intravascular ultrasound RDN offers potential benefits in preserving the vascular endothelium of renal arteries of varying morphologies. The ultrasound-based Paradise system has been approved by the FDA and CE and is currently used in clinical practice across the United States and Europe as an adjunct therapy for uncontrolled hypertension [17].
3. Chemical denervation induces relaxation of renal nerves, thereby attenuating sympathetic nervous system activity and subsequently lowering BP. Agents like alcohol, vincristine, and guanethidine are utilized to target and impair renal nerves, leading to demyelination. In comparison to RF ablation, chemical denervation produces more extensive lesions and markedly decreases sympathetic nerve function [7]. Chemical RDN remains investigational and has not yet received FDA or CE approval.
4. High-intensity focused ultrasound (HIFU) employs piezoelectric transducers to concentrate high-frequency sound waves, producing thermal energy that enables the precise ablation of targeted tissues. As a noninvasive modality, HIFU permits accurate treatment of deep-seated structures without the need for contrast agents or ionizing radiation [20]. The procedure is readily applicable in standard clinical settings, thereby enhancing patient accessibility, accelerating recovery, and reducing healthcare expenditures. Although HIFU is associated with potential thermal effects, including injury to adjacent organs or tissues, skin burns, and cavitation, these risks are generally low and can be effectively mitigated through appropriate instrumentation and experienced clinical operators [21]. Clinically, HIFU is widely applied in tumor ablation [22]; however, its role in RDN remains experimental and requires further validation through clinical trials.

To facilitate comparison, Table 2 summarizes the key characteristics, mechanisms of action, clinical approval status, and major clinical trials associated with the four principal RDN modalities.

## 4. RDN Research Status

In order to evaluate the efficacy and safety of RDN, both domestic and international scholars have conducted numerous clinical trials (shown in Table 3).

### 4.1. SYMPLICITY: Pioneering RDN Trials and Lessons From Methodological Flaws

The SYMPLICITY HTN-1 trial (2009) initially enrolled 50 patients and reported a mean office BP reduction of 27/17 mmHg at 12 months [23]. An extended follow-up involving 88 patients showed a sustained reduction of 32.0/14.4 mmHg at 36 months [16], suggesting long-term efficacy. However, the absence of a control group and blinding limited the validity of the findings.

The SYMPLICITY HTN-2 trial (2010) introduced a control arm using baseline antihypertensive therapy and demonstrated a significantly greater office BP reduction in the RDN group (32/12 mmHg) compared to controls [24]. However, the study was limited by the absence of a sham procedure, lack of blinding, small sample size, and limited ambulatory BP assessment.

The SYMPLICITY HTN-3 trial (2014) addressed prior limitations with a multicenter, randomized, single-blind, sham-controlled design. However, at 6 months, the BP reduction was not significantly different between groups (SBP change: −14.1 ± 23.9 vs. −11.7 ± 25.9 mmHg; between-group difference: −2.4 mmHg, *p* = 0.26) [25]. Possible contributors to the null findings included suboptimal first-generation catheter performance, inconsistent denervation technique, and poor medication adherence [32]. Subsequent clinical studies [33] also reported negative results following the SYMPLICITY HTN-3 trial.

### 4.2. SPYRAL: Revalidating RDN Through Rigorous Design and Modern Technology

Since 2017, RDN research has adopted multielectrode catheters capable of simultaneous four-quadrant ablation [34], improving procedural consistency and denervation efficacy.

The SPYRAL HTN-OFF MED trial (2017) improved upon SYMPLICITY HTN-3 by enrolling untreated patients with mild to moderate hypertension, adopting a sham-controlled, single-blind design, and employing multielectrode catheters with enhanced circumferential ablation coverage to minimize confounding and improve efficacy. At 3 months, RDN significantly reduced 24-h SBP and DBP compared to sham (−5.0 and −4.4 mmHg, respectively), demonstrating the antihypertensive potential of RDN independent of pharmacotherapy [26]. However, its limited sample size constrained broader applicability.

The subsequent SPYRAL HTN-OFF MED Pivotal trial (2020) expanded on this design with a larger sample size (*n* = 331), confirming significant 3-month reductions in 24-h SBP (−4.0 mmHg, 95% CI: −6.2 to −1.8) and DBP (−3.1 mmHg, 95% CI: −4.6 to −1.7) in the RDN group compared to sham [28]. These findings reinforced the reproducibility and statistical robustness of RDN in medication-free populations. Nonetheless, the applicability of results remained limited by the artificial setting of drug withdrawal, which does not reflect typical clinical practice.

In conjunction with the SPYRAL HTN-OFF MED trial, the SPYRAL HTN-ON MED trial (2018) provided complementary evidence supporting the antihypertensive efficacy of RDN in both untreated and medicated patients. The SPYRAL HTN-ON MED trial confirmed the efficacy of RDN across different treatment settings. In patients on stable antihypertensive therapy (*n* = 80), RDN achieved additional reductions in 24-h SBP (−7.0 mmHg) and DBP (−4.3 mmHg) at 6 months [27]. Long-term results at 36 months showed sustained BP lowering (SBP −10 mmHg; DBP −5.9 mmHg) [35], reinforcing the durability and clinical utility of RDN alongside pharmacologic treatment.

### 4.3. RADIANCE: Ultrasound-Guided RDN and Stratified Approaches to Hypertension

While the majority of earlier clinical trials focused on RF-based RDN, ultrasound-guided RDN has emerged as a promising alternative, offering potential advantages in terms of circumferential energy delivery and more consistent ablation of perivascular sympathetic nerves. The RADIANCE trial program provides pivotal evidence supporting the efficacy of this modality across different hypertensive phenotypes.

The RADIANCE-HTN SOLO trial (2018) enrolled patients (*n* = 146) with mild to moderate hypertension who remained hypertensive after a 4-week drug washout, thereby minimizing medication-related confounding [36]. The trial demonstrated that ultrasound RDN reduced daytime ambulatory SBP by 8.5 mmHg at 2 months, 6.3 mmHg more than sham (*p* = 0.0001). Although the design minimized drug-related confounding, its short follow-up and lack of background antihypertensive therapy limit generalizability to routine clinical practice.

The RADIANCE-HTN TRIO trial (2021) targeted patients with confirmed resistant hypertension already receiving standardized triple fixed-dose therapy. By reducing pill burden and ensuring high baseline adherence, the study provided a controlled setting to evaluate add-on RDN effects. The results revealed an additional 4.5 mmHg reduction in daytime ambulatory SBP in the RDN group versus sham (95% CI: −8.5 to −0.3; *p* = 0.022), indicating incremental benefit even in pharmacologically optimized patients [29]. However, applicability to patients beyond those on standardized triple therapy is constrained, and similar to RF RDN, the absence of reliable intraoperative indicators of denervation efficacy remains a major limitation.

### 4.4. SMART Trial: Precision-Guided RDN via Real-Time Nerve Mapping

The SMART trial introduced a novel mapping-guided RDN strategy that incorporated real-time electrical stimulation to identify functionally active renal sympathetic nerves. Only sites showing a systolic BP rise of ≥ 5 mmHg were ablated, with postablation stimulation used to confirm efficacy. This prospective, multicenter, single-blind RCT enrolled 220 patients and evaluated two primary endpoints at 6 months: (1) the proportion of patients achieving office SBP < 140 mmHg and (2) changes in a drug composite index reflecting both medication class and dosage [30].

While the office SBP control rate was similar between groups (95.4% vs. 92.8%, *p* = 0.429), the msRDN group required significantly fewer antihypertensive drugs (Δ = −3.25, *p* = 0.003). The comparable 24-h SBP reductions (−10.8 vs. −10.0 mmHg, *p* > 0.05) indicated that the primary benefit lay in reduced drug dependence. High adherence rates (> 90%) verified by LC-MS/MS reinforce the reliability of these findings [31].

Compared with prior RDN trials (SPYRAL and RADIANCE), which relied on anatomical assumptions and nontargeted ablation, the SMART trial uniquely incorporated functional nerve mapping to guide ablation based on individual BP responses to stimulation. This real-time physiological feedback approach enhanced procedural specificity and theoretically improved denervation precision.

## 5. Limitations of RDN Therapy

The SYMPLICITY HTN-3 trial yielded contradictory findings regarding the efficacy of intravascular catheter RDN in reducing SBP compared to sham operation controls [25]. This discrepancy highlights the technical challenges of catheter-based RDN therapy and RF energy delivery, including uneven ablation, limited lesion coverage, difficulty accessing small renal arteries, and potential local endothelial damage [21]. Additionally, there are few effective predictors for RDN outcomes, limited comparisons of ablation techniques, insufficient long-term studies on antihypertensive efficacy and safety, unclear surgical guidelines for moderate to severe renal insufficiency, and a lack of trials on hypertensive complications [37].

As a minimally invasive procedure, RDN carries the risk of various complications, including hematoma formation at the access site, renal artery stenosis, pseudoaneurysm development, and contrast-induced acute kidney injury [19, 23, 36]. While these adverse events are infrequent, they may manifest during or immediately following the intervention. A meta-analysis of 50 studies on RDN therapy found that 26 out of 5769 patients experienced renal artery issues, with 79% of these events occurring within a year [38]. Furthermore, research indicates that RDN may impact renal function in certain individuals. A study conducted by Mahfoud et al. [39] demonstrated that over a 36-month period, individuals without chronic kidney disease exhibited a decline in kidney function of 7.1 mL/(min·1.73 m^2^), whereas those with chronic kidney disease exhibited a decline of 3.7 mL/(min·1.73 m^2^).

## 6. Advantages of HIFU

HIFU has shown efficacy in tissue ablation [22] across various clinical contexts. HIFU, a noninvasive ultrasound modality, can selectively ablate the outer layer of the artery without affecting the inner lining of the renal artery. Prior to administering HIFU therapy, a personalized treatment plan is devised based on computed tomography (CT) imaging, facilitating the successful execution of RDN while mitigating harm to adjacent organs. This approach minimizes vascular damage, lowers the likelihood of postoperative renal artery stenosis, and mitigates the invasive risks associated with femoral artery puncture and catheter procedures with minimal reliance on contrast agents or exposure to radiation. And ultrasound exhibits superior tissue penetration capabilities, thereby limiting the efficacy of RF catheter ablation when the depth of tissue affected exceeds 7 mm [6]. HIFU has the capability to deliver ultrasound energy precisely to a focal point located within 10 mm of the bifurcation of the renal artery, resulting in a localized temperature increase of 50°C–75°C [21, 22] and increased ablation depth. Furthermore, this technique can be utilized in a standard clinical setting without the need for interventional equipment. This modality offers a brief treatment duration, thereby enhancing patient accessibility and expediting recovery times, while also contributing to a reduction in long-term healthcare costs [40]. Overall, the advantages of HIFU can compensate for the shortcomings observed in the SYMPLICITY HTN-3 trial. These shortcomings include operator experience, inadequate depth of the RF ablation catheter for the distal renal artery, insufficient ablation depth, and operational deviations associated with the single-electrode ablation catheter [41]. Currently, HIFU has received FDA approval for applications such as tumor resection in cardiac tissue and ablation of internal soft tissue. The method involves the use of multiple piezoelectric transducers to focus high-frequency sound waves, inducing a thermal effect that leads to precise ablation of the intended tissue. While thermal injury to adjacent organs and tissues, skin damage at the interface with the treatment device, and discomfort in the back are recognized as potential adverse outcomes of HIFU, the likelihood of experiencing these effects is minimal and can be mitigated through proper calibration of equipment and training of healthcare providers [42]. Two existing systems, namely, the Medical Surround Sound system (Kona Medical Inc., Bellevue, Washington, United States) and the Model-JC HIFU Oncology Treatment System, utilize external ultrasound technology to precisely monitor the position and motion of the renal artery and effectively administer focused ablation therapy to the targeted nerve surrounding the renal artery, eliminating the necessity for a catheter [42].

## 7. Preclinical Study of HIFU

Several preclinical studies were conducted before human trials began, including assessing the feasibility and safety of the Surround Sound system, determining optimal targeting and therapeutic dosage levels, and examining lesion patterns. Wang and colleagues studied the potential of HIFU for noninvasive renal sympathetic nerve ablation. Canine subjects were utilized in the study, utilizing the Model-JC HIFU tumor treatment system. Under the guidance of Doppler flow imaging, therapeutic ablation (250 W × 2 s) was conducted on 18 healthy dogs in the ablation group. Both renal nerves were ablated using HIFU (36.3 ± 2.8 HIFU dose per animal), with an average duration of 27.4 min. In the sham group, five dogs underwent a similar surgical procedure without acoustic therapy. Posttreatment, dogs in the ablation group exhibited a 15.9/13.6 mmHg reduction in BP after 28 days, accompanied by histological signs of neuron and Schwann cell necrosis, confirming complete renal nerve destruction [43]. A porcine study examined both the safety and the efficacy of MR-guided HIFU after unilateral RDN in five animals [44]. An assessment of norepinephrine levels in the kidney medulla was performed after the animals were sacrificed 6–9 days posttreatment. Ablation significantly reduced (*p* value = 0.04) the cross-sectional area of nerve bundles between treated and untreated renal arteries in all animals with treated nerves presenting increased cellular infiltrate and fibrosis, and there was no indication of tissue damage in arterial walls. Based on the results of preclinical studies, few significant adverse events or complications were observed. Overall, preclinical animal studies have substantiated the efficacy and safety of HIFU for RDN, thereby facilitating the commencement of clinical trials.

## 8. Clinical Study of HIFU

To establish the safety and feasibility of HIFU for clinical use in RDN, a series of trials have been conducted over the past decade (shown in Table 4).

### 8.1. WAVE I–III Trials: Early Stage Feasibility and Technical Iteration

The WAVE trials represented a phased effort to develop and refine externally focused HIFU as a noninvasive approach for RDN in resistant hypertension.

In WAVE I, 24 patients with a mean baseline BP of 190/100 mmHg and an average of 4.5 antihypertensive medications underwent bilateral HIFU-RDN under general anesthesia. Eighteen ablation lesions were delivered per side using an intravascular targeting catheter. After 6 months, office SBP decreased by 29 mmHg on average, with no serious adverse events reported [45]. However, approximately 50% of patients experienced transient back pain attributed to high energy delivery.

WAVE II enrolled 18 similar patients and optimized the treatment protocol to reduce ablation time (approximately 2.8 min per side) and energy dose. The mean SBP reduction at 6 weeks was 18 mmHg [42], consistent with WAVE I. Improved energy modulation led to fewer MRI-detected tissue abnormalities, and no motor or sensory deficits were observed. Again, approximately 50% reported mild back pain that resolved spontaneously.

WAVE III eliminated the need for catheter-based targeting by implementing real-time external Doppler ultrasound imaging [46]. Initially, five patients underwent catheter-guided ablation for validation, followed by 22 patients who received fully noninvasive image-guided HIFU treatment. Office SBP reductions averaged 29.6/11.8 mmHg at 12 weeks and 28.6/11.3 mmHg at 12 months. At 6 and 12 months, 75%–77% of patients achieved ≥ 10 mmHg reductions in SBP, with 55%–56% achieving ≥ 20 mmHg. Approximately 41% experienced mild back pain, resolving within 48 h. No serious complications were reported [47].

Collectively, the WAVE I–III trials demonstrated the feasibility, safety, and antihypertensive potential of externally focused HIFU-RDN, with progressive improvements in targeting accuracy and procedural tolerability. However, the lack of sham control and significant variability in BP response limit the interpretability and generalizability of these findings.

### 8.2. WAVE IV: Sham-Controlled Validation Reveals Efficacy Uncertainty

To address the limitations of prior WAVE I–III trials lacking placebo control, WAVE IV employed a randomized, double-blind, sham-controlled design with a noninvasive sham procedure and extended follow-up. Among 81 patients, no significant differences in office BP or ambulatory BP reductions were observed between the RDN and sham groups at 12 or 24 weeks. Office SBP decreased by 13.2 ± 20 versus 18.9 ± 14 mmHg at 12 weeks (*p* = 0.181) and 12.8 ± 26 versus 23 ± 20 mmHg at 24 weeks (*p* = 0.133). Ambulatory SBP and DBP showed no significant differences in reduction between the two groups [48]. These findings failed to demonstrate a clear antihypertensive advantage of extracorporeal focused ultrasound over placebo, highlighting the need for further validation.

WAVE IV trials failed to demonstrate a superior BP-lowering effect of extracorporeal HIFU-based RDN compared to sham control, likely due to high placebo response, heterogeneous patient baseline, and the lack of intraprocedural markers of denervation success. These limitations underscore the need for refined patient selection, better energy targeting, and procedural verification in future studies.

### 8.3. The Model-JC System: Complementary Evidence for Noninvasive RDN Feasibility

The Model-JC system is a noninvasive RDN platform that combines real-time B-mode ultrasound with a HIFU transducer, enabling precise visualization and targeting of renal sympathetic nerves. Its coaligned imaging allows adjustment for anatomical variability and respiratory motion, while digital treatment planning ensures millimeter-level focal accuracy. Optimized energy dosing, supported by cooling and monitoring, enhances ablation depth.

In clinical evaluations involving 47 patients, the Model-JC system achieved substantial and statistically significant BP reductions. At 1, 3, and 6 months, mean 24-h ambulatory BP decreased by −13.1/−7.6, −14.9/−9.0, and −11.4/−4.8 mmHg, respectively (all *p* < 0.001); corresponding office BP reductions were −25.6/−10.2, −29.9/−12.2, and −29.2/−11.2 mmHg (all *p* < 0.001), indicating a robust and sustained antihypertensive effect [49].

The Model-JC HIFU trials present notable methodological and technological advantages over the WAVE IV trial. While WAVE IV enrolled a heterogeneous population with office SBP ≥ 160 mmHg and 24-h SBP ≥ 135 mmHg despite triple therapy, it included patients with advanced arterial stiffness and severe hypertension, potentially blunting the response to sympathetic denervation [50]. In contrast, the Model-JC studies likely applied stricter screening to exclude secondary causes and focus on patients with sympathetic overactivity, ensuring a more homogeneous and responsive cohort.

Technologically, the Model-JC system integrates real-time B-mode ultrasound with the HIFU transducer, enabling direct visualization of renal arteries and dynamic adjustment for respiratory or anatomical variability. This coaligned imaging provides more accurate, reproducible targeting than WAVE IV's external Doppler guidance, which lacked real-time anatomical confirmation. Additionally, the system allows digital control of sonication parameters and precise energy delivery, optimized through preclinical studies.

However, the current Model-JC platform has intrinsic limitations. As a fixed-focus, single-element transducer, it suffers from energy attenuation through overlying tissues and lacks the flexibility to steer or shape the beam. Variations in renal artery depth or anatomical barriers (e.g., ribs and bowel gas) may hinder effective targeting, requiring manual probe repositioning and increasing the risk of incomplete denervation. These factors limit its adaptability and procedural efficiency. Future upgrades using phased-array focused ultrasound offer a promising solution [51]. This technology enables electronic beam steering and multispot ablation without moving the transducer, allowing circumferential and longitudinal nerve coverage in a single session. It can dynamically adjust for tissue heterogeneity, minimize off-target heating, and improve safety, comfort, and procedural speed. This technology is technically feasible, as similar phased-array HIFU systems are already in clinical use for other targets [52, 53]. Incorporating such advancements could further enhance the clinical utility of noninvasive RDN.

## 9. Summary and Prospect

Several future directions are emerging in the field of RDN, focusing on optimizing efficacy, precision, and safety. Ongoing research efforts include the refinement of patient selection protocols to more accurately identify individuals most likely to benefit from RDN [54]. These efforts are complemented by large-scale multicenter clinical trials and long-term follow-up studies, which aim to assess the durability of BP control and to identify any potential late-onset complications [55]. The findings from these studies will be critical in guiding future clinical applications and defining the broader role of RDN in hypertension management.

Another promising direction is the exploration of combination therapy strategies. By integrating RDN with conventional antihypertensive treatments, such as pharmacological agents and lifestyle interventions, researchers hope to develop more comprehensive and sustained approaches to BP management.

In parallel, technological innovation is propelling the development of HIFU as a noninvasive RDN modality. Current research initiatives are exploring the design of new phased array transducers, utilizing three-dimensional anatomical modeling and simulation techniques to optimize acoustic targeting and energy delivery to renal sympathetic nerves. These technical improvements are being validated through preclinical experiments and are expected to inform the next generation of HIFU-based systems for hypertension therapy.

In summary, ongoing research reflects a growing interest in RDN as a component of personalized medicine. While promising, these innovations remain under investigation; further evidence is required before routine clinical implementation.

## Figures and Tables

**Table 1:** Comparison of ACC/AHA versus ESH/ESC guidelines.

	**ACC/AHA (2017) guidelines [** 4 **]**	**ESC/ESH(2018) guidelines [** 5 **]**
Hypertension diagnosis	SBP ≥ 130 or DBP ≥ 80 mmHg (formerly SBP ≥ 140 or DBP ≥ 90 mmHg)	SBP ≥ 140 or DBP ≥ 90 mmHg
Normal BP range	Normal BP < 120/80 mmHg Elevated BP 120–129/< 80 mmHg	Optimal BP < 120/80 mmHg Normal BP 120–129/80–84 mmHg High normal 130–139/85–89 mmHg
Stage 1 hypertension (mild)	Stage 1: SBP 130–139 or DBP 80–89 mmHg	Grade 1: SBP 140–159 or DBP 90–99 mmHg
Stage 2 hypertension (moderate/severe)	Stage 2: SBP ≥ 140 or DBP ≥ 90 mmHg	Grade 2: SBP 160–179 or DBP 100–109 mmHg Grade 3: SBP ≥ 180 or DBP ≥ 110 mmHg
Resistant hypertension	Blood pressure that remains above 130/80 mmHg despite concurrent use of ≥ 3 antihypertensive medications of different classes at optimal doses (one should be a diuretic), or BP that is controlled to goal only with ≥ 4 medications	Blood pressure cannot be reduced to < 140/90 mmHg despite appropriate triple-drug therapy (of different classes, at optimal doses)
Estimated hypertension prevalence	About 45.6% of US adults qualify as having hypertension—a rise from 31.9% under the older 140/90 mmHg definition	Roughly 30%–45% of adults in Europe have hypertension

**Table 2:** RDN technique summary.

**Technique**	**Key features**	**Major clinical trials**	**Clinical use status**
Radiofrequency RDN	Thermal ablation via catheter-mounted electrodes; spiral or multielectrode tips target adventitial nerves	SYMPLICITY HTN-1/2/3, SPYRAL HTN-OFF MED, SPYRAL HTN-ON MED	FDA approved (Symplicity Spyral); CE marked; used in select centers for resistant hypertension
Ultrasound-based RDN	Circumferential energy delivery via intravascular balloon catheter; preserves endothelium	RADIANCE-HTN SOLO, RADIANCE-HTN TRIO	FDA approved (Paradise); CE marked; adopted in EU/US centers for adjunct therapy
Chemical RDN	Neurolytic agents injected perivascularly to chemically ablate renal nerves	TARGET BP I, TARGET BP OFF-MED	Investigational; not FDA or CE approved; limited to trials and feasibility studies
HIFU	Noninvasive external ultrasound focuses energy on renal arteries without catheterization	WAVE I–IV	Clinically approved and widely used for tumor treatment; remains investigational for RDN and requires further clinical validation

**Table 3:** RDN clinical trials.

**Date**	**Clinical trial**	**RDN catheter**	**RDN**	**Control**	**Primary endpoint**	**Primary endpoint situation**
2009	SYMPLICITY HTN-1 [23]	SYMPLICITY FLEX	153	NA	12-month office SBP	RDN treatment group: −22 mmHg, *p* < 0.001
2010	SYMPLICITY HTN-2 [24]	SYMPLICITY FLEX	52	54	6-month office SBP	RDN treatment group: −32 ± 23 mmHg Control group: 1 ± 21 mmHg, *p* < 0.0001
2014	SYMPLICITY HTN-3 [25]	SYMPLICITY FLEX	364	171	6-month office SBP	RDN treatment group: −14.1 ± 23.9 mmHg Sham operation control group: −11.7 ± 25.9 mmHg, for a difference of −2.39 mmHg, *p* = 0.26
2017	SPYRAL HTN-OFF MED [26]	SYMPLICITY SPYRAL	166	165	3-month 24 h by ambulatory SBP	24-h SBP: −5.0 mmHg (95% CI −9.9 to −0.2; *p* = 0.0414) 24-h DBP: −4.4 mmHg (95% CI −7.2 to −1.6; *p* = 0.0024)
2018	SPYRAL HTN-ON MED [27]	SYMPLICITY SPYRAL	38	42	6-month 24 h by ambulatory SBP	24-h SBP difference: −7.4 mmHg (95% CI −12.5 to −2.3; *p* = 0.0051) 24-h DBP difference: −4.1 mmHg (95% CI −7.8 to −0.4; *p* = 0.0292)
2018	RADIANCE-HTN SOLO [19]	PARADISE	73	73	2-month daytime SBP by ambulatory SBP	RDN treatment group: −8.5 ± 9.3 mmHg Sham operation control group: −2.2 ± 10.0 mmHg Baseline-adjusted difference between groups: −6.3 mmHg (95% CI −9.4 to −3.1, *p* = 0.0001)
2020	SPYRAL HTN-OFF MED Pivotal [28]	SYMPLICITY SPYRAL	166	165	3-month 24 h by ambulatory SBP	The 24-h SBP difference between the two groups was −3.9 mmHg (95% CI −6.2 to −1.6, *p* = 0.005)
2021	RADIANCE-HTN TRIO [29]	PARADISE	69	67	2-month daytime SBP by ambulatory SBP	RDN treatment group: −8.0 mmHg Sham operation control group: −3.0 mmHg, *p* = 0.022
2024	SMART [30, 31]	SyMapCath I and SYMPIONEER	110	110	The control rate of office SBP to achieve level of < 140 mmHg and change in the drug composite index of antihypertensive medications at 6 months	BP control: The msRDN group achieved a control rate of 95.4%, while the sham group achieved 92.8% (*p* = 0.429) Drug index: The msRDN group was superior to sham group (Δ = −3.25, 95% CI −5.56 to −0.94, *p* = 0.003) 24-h ambulatory SBP: msRDN group reduced by 10.8 ± 14.1 vs. 10.0 ± 14.0 mmHg in sham group (*p* < 0.001 from baseline; *p* > 0.05 between groups)

**Table 4:** Summary of HIFU-based RDN trials.

**Trial**	**Trial design**	**Patient selection**	**Results**	**Key limitations**
WAVE I	Single-arm study using intra-arterial catheter-guided HIFU	Mean age: 60 Resistant HTN Included patients on multiple drugs	*Office BP* decreased by approximately 29 mmHg	*Design*: No control or blinding; placebo effect cannot be ruled out *Device*: Invasive catheter and fixed depth reduced treatment precision
WAVE II	Single-arm trial with catheter guidance	Mean age: 60 Resistant HTN Patients on 4–5 antihypertensive drugs	*Office BP* reduced by about 18 mmHg at 6 months; 75% of patients achieved > 10 mmHg SBP drop	*Design*: No randomization or blinding, single arm *Patient selection*: Possible inclusion of patients less likely to respond; medication adjustments during trial were not fully controlled *Device*: External HIFU still required invasive imaging for targeting. Energy delivery depth fixed
WAVE III	Single-arm study using intra-arterial catheter-guided HIFU	Mean age: 60 Resistant HTN Patients on 4–5 antihypertensive drugs	*Office BP* reductions ranged from 25 to 30/10 mmHg *24-h SBP* dropped by ~10 mmHg at 6 months	*Design*: No sham or control. Small sample size *Patient selection*: Some potentially with advanced arterial disease *Device*: Limited targeting accuracy with external ultrasound guidance
WAVE IV	Phase II randomized, sham-controlled, double-blind trial. Stopped early (*n* = 81) for futility	Wide age range True resistant HTN	*Office SBP* dropped by 13.2 (RDN) vs. 18.9 mmHg (sham) (*p* = 0.133) *24-h SBP*: 7.1 vs. 5.9 mmHg (*p* = 0.77)	*Design*: Sample size cut nearly in half due to early termination *Patient selection*: Broad inclusion criteria enrolled less-responsive patients *Device*: External HIFU dose may have been insufficient in humans
Model-JC	Single-arm pilot study using the Model-JC HIFU system	Age not reported Resistant HTN Required stable medication for ≥ 4 weeks. Patients were on various standard drugs	*Office BP* declined by ~20 mmHg *24-h BP* dropped by 13/8 mmHg at 6 months (*p* < 0.01)	*Design*: Single-center, unblinded study with no control group. Small sample size *Device*: Fixed-focus design causes energy loss and inflexible targeting, with anatomical variations risking incomplete denervation

## Data Availability

Data sharing is not applicable to this article as no new data were created or analyzed in this study.
